# More Human BM-MSC With Similar Subpopulation Composition and Functional Characteristics Can Be Produced With a GMP-Compatible Fabric Filter System Compared to Density Gradient Technique

**DOI:** 10.3389/fcell.2021.638798

**Published:** 2021-03-29

**Authors:** Gabriele Spohn, Anne-Sophie Witte, Anja Kretschmer, Erhard Seifried, Richard Schäfer

**Affiliations:** Institute for Transfusion Medicine and Immunohematology, Goethe University Hospital, German Red Cross Blood Service Baden-Württemberg—Hessen gGmbH, Frankfurt am Main, Germany

**Keywords:** mesenchymal stromal cell, production, heterogeneity, subpopulation, function, GMP

## Abstract

**Background:**

Mesenchymal stromal cells (MSCs), multipotent progenitors that can be isolated from a variety of different tissues, are becoming increasingly important as cell therapeutics targeting immunopathologies and tissue regeneration. Current protocols for MSC isolation from bone marrow (BM) rely on density gradient centrifugation (DGC), and the production of sufficient MSC doses is a critical factor for conducting clinical MSC trials. Previously, a Good Manufacturing Practice (GMP)–compatible non-woven fabric filter device system to isolate MSCs was developed to increase the MSC yield from the BM. The aim of our study was to compare high-resolution phenotypic and functional characteristics of BM-MSCs isolated with this device and with standard DGC technology.

**Methods:**

Human BM samples from 5 donors were analyzed. Each sample was divided equally, processing by DGC, and with the filter device. Stem cell content was assessed by quantification of colony-forming units fibroblasts (CFU-F). Immunophenotype was analyzed by multicolor flow cytometry. *In vitro* trilineage differentiation potential, trophic factors, and IDO-1 production were assessed. Functionally, immunomodulatory potential, wound healing, and angiogenesis were assayed *in vitro*.

**Results:**

The CFU-F yield was 15-fold higher in the MSC preparations isolated with the device compared to those isolated by DGC. Consequently, the MSC yield that could be manufactured at passage 3 per mL collected BM was more than 10 times higher in the device group compared to DGC (1.65 × 10^9^ vs. 1.45 × 10^8^). The immunomodulatory potential and IDO-1 production showed donor-to-donor variabilities without differences between fabric filter-isolated and DGC-isolated MSCs. The results from the wound closure assays, the tube formation assays, and the trilineage differentiation assays were similar between the groups with respect to the isolation method. Sixty-four MSC subpopulations could be quantified with CD140a^+^CD119^+^CD146^+^ as most common phenotype group, and CD140a^+^CD119^+^CD146^+^MSCA-1^–^CD106^–^CD271^–^ and CD140a^+^CD119^+^CD146^–^MSCA-1^–^CD106^–^CD271^–^ as most frequent MSC subpopulations. As trophic factors hepatocyte growth factor, epidermal growth factor, brain-derived neurotrophic factor, angiopoietin-1, and vascular endothelial growth factor A could be detected in both groups with considerable variability between donors, but independent of the respective MSC isolation technique.

**Conclusion:**

The isolation of MSCs using a GMP-compatible fabric filter system device resulted in higher yield of CFU-F, producing substantially more MSCs with similar subpopulation composition and functional characteristics as MSCs isolated by DGC.

## Introduction

Mesenchymal stromal cells (MSCs) feature, via paracrine signaling, direct cell-to-cell contact, and/or extracellular vesicles delivery, powerful immunomodulation and tissue regeneration properties (Fontaine et al., 2016; Phinney and Pittenger, 2017). This makes them promising candidates for the development of cell therapeutics tackling immunopathologies or autoimmune diseases (Trounson and McDonald, 2015; Leyendecker et al., 2018). Specifically, they have been shown to ameliorate refractory graft-versus-host disease, to support the recovery of organ and tissue function, e.g., after stroke, myocardial infarction, or spinal cord injury, or to repair skin wounds (Hu et al., 2018; Bieback et al., 2019b). However, the clinical impact of MSC applications has been inconsistent (Trounson and McDonald, 2015). The reasons for these observations have not been elucidated completely, but the considerable heterogeneity of MSC preparations may be a relevant contributor. The composition of MSC preparations varies between donors (Siegel et al., 2013), and there is emerging evidence linking MSC subpopulation phenotypes to their therapeutic potential (Rennert et al., 2016; Khong et al., 2019; Kuci et al., 2019; Danielyan et al., 2020).

Moreover, the isolation procedure and manufacturing conditions of MSCs may additionally impact their function (Astori et al., 2016). To date, the main sources for MSC therapeutics manufacture are bone marrow (BM) and adipose tissue (Schäfer et al., 2016). The current standard technology for MSC isolation from the BM is the density gradient centrifugation (DGC) producing a mononuclear cell–enriched fraction that is plated into culture vessels where, after removal of the non-adherent cells, the adherent MSCs are further subcultured (Dominici et al., 2006; Siegel et al., 2013). Other approaches are direct BM plating (Mareschi et al., 2012) or removing red blood cells from the BM aspirate with ammonium chloride treatment that can be more effective for BM-MSC isolation than DGC technology (Horn et al., 2008). The goal of this *ex vivo* culture is to generate a sufficient MSC yield to formulate enough doses to serve clinical trials. This can be achieved by “classical” 2D (multilayer) culture flasks or 3D systems such as roller bottles or bioreactors where several billions of MSCs can be produced under the requirements of Good Manufacturing Practice (GMP) (Mizukami and Swiech, 2018; Bieback et al., 2019b). Ideally, MSCs *ex vivo* expansion shall be limited to avoid replicative senescence that can lead to genetic instability and decrease their immunomodulatory potential by proteasomal indoleamine 2,3-dioxygenase (IDO) degradation (Galipeau, 2013; Loisel et al., 2017).

Thus, sufficient starting material, combined with an optimized MSC isolation technique, is needed to produce the required MSC doses at minimal *ex vivo* culturing time. To address this issue, a non-woven fabric filter device was developed for the isolation of MSCs from the BM based on a combination of retention via filter effect and adherence to the fibers (“adherent trapping”) (Ito et al., 2010). Indeed, previous studies showed that this device could not only reduce the required open steps compared to DGC, but also increase the numbers of MSCs that could be harvested for *ex vivo* culture (Ito et al., 2010). However, it has been unknown if filtering of the BM cells (BMCs) through this device would impact the BM-MSC heterogeneity and/or their function. Here we analyzed BM-MSCs that were isolated with the device or with the DGC technology for their *ex vivo* manufacturing yields and their subpopulation compositions, as well as for their functions.

## Materials and Methods

### Bone Marrow Donors

Human BM samples (44–48 mL each) were collected from 5 healthy donors (1 female and 4 male donors, aged 21–33 years) after informed donor consent and were subsequently processed and analyzed.

The study was approved by the ethics committee of the Goethe University Medical Center, Frankfurt, Germany (ethics committee approval #383/13).

### BM Processing

BM, anticoagulated with 50 U/mL Heparin (Ratiopharm, Ulm, Germany) and 0.15 mL ACD-A/mL (Fresenius, Bad Homburg v.d.H., Germany), was processed directly after collection. Each sample was divided equally with a minimum of 22 mL per isolation method as prescribed by the protocol, followed by processing one-half with the filter device (CellEffic BM; Kaneka Corporation, Japan) and the other half with DGC.

### Density Gradient Centrifugation

BMCs were isolated by DGC on lymphocyte separation medium (Lonza, Basel, Switzerland), followed by erythrocyte lysis (red blood cell lysing buffer; Sigma–Aldrich, St Louis, MO, United States) and resuspended in cell culture medium, composed of Dulbecco modified eagle medium (DMEM)–low glucose (Thermo Fisher Scientific, Waltham, MA, United States), 10% fetal bovine serum (FBS) (Wako Chemicals GmbH, Neuss, Germany), and 1% penicillin–streptomycin (Thermo Fisher Scientific).

### Device

Isolation of MSCs with the BM filter device was performed according to the manufacturer’s protocol. The main component of the device is a filter composed of rayon and polyethylene with fibers featuring a diameter of 15 μm and a contact angle of 20° (Ito et al., 2010). MSCs can better adhere to hydrophilic/rough surfaces than to hydrophobic/smooth surfaces (Ito et al., 2010). Thus, the highly hydrophilic surface of the fibers supports adhesion of the MSCs, which can be eluted thereafter (“adherent trapping” mechanism) (Ito et al., 2010). Briefly, the device was primed with sterile saline and filled with the collected BM. After washing with sterile saline the filter-trapped BM-MSCs were harvested into a closed collection bag by back flushing the device with 50 mL cell culture medium.

### Colony-Forming Unit Fibroblasts

To determine the progenitor cell content, the numbers of colony-forming unit fibroblasts (CFU-F) were assessed at passage (P) 0. For both isolation methods, the volume of BMC suspension to be plated was determined by protocol using the following formula:

Plating volume = volume of BMC suspension ÷ volume of processed BM × 0.3528

The volume was adjusted to 4 mL; cells were seeded into 6 cm^2^ cell culture dishes and maintained at 37°C in humidified atmosphere with 5% CO_2_ for 9–11 days. CFU-F were stained with 0.5% crystal violet solution (Sigma–Aldrich) and counted, and the number of colonies per mL of BM was calculated using the following equation:

CFU-F/mL = colony number × 2.8

### BM-MSC Culture

For both isolation methods, the volume of BMC suspension to be plated was determined by protocol using the following formula:

Plating volume = 2.7 × 10^5^ × 58.1 ÷ concentration of BMC suspension

resulting in a plating density of 2.85 × 10^5^ cells/cm^2^.

The volume was adjusted to 10 mL; cells were seeded into 10 cm^2^ cell culture dishes and maintained in cell culture medium at 37°C in humidified atmosphere with 5% CO_2_. When the BM-MSCs reached subconfluency, they were detached using 0.25% trypsin-EDTA (Thermo Fisher Scientific) and either cryopreserved (DMEM, 35% FBS, 5% DMSO [Sigma–Aldrich]) for functional assays or seeded at a density of 1,000 cells/cm^2^. The BM-MSCs were cultured up to P3.

### Growth Kinetics

To assess the *ex vivo* growth kinetics of the BM-MSCs, the population doubling time (PDT) and the cumulative population doubling level (cPD) were determined.

The PDT in exponential growing cultures can be calculated by the following formula:

PDT = ln(2) ÷ growth rate

where growth rate is ln (*N*_(_*_*t*_*_)_ ÷ *N*_(0)_) ÷ t, *N*_(_*_*t*_*_)_ is cells at time *t*, *N*_(0)_ is cells at time 0, and *t* is time in culture.

Thus, the PDT was calculated by the following equation:

PDT = *t* × ln(2) ÷ ln(*N*_(_*_*t*_*_)_ ÷ *N*_(0)_) = culture time × ln (2) ÷ ln (cell number_*harvest*_ ÷ cell number_*seeded*_)

The population doubling level depends on the relationship between the number of cells initially in culture (*N*_0_) and the cell number after a period of exponential growth (N) (Madigan et al., 2000). The relationship of these parameters can be described as *N* = *N*_0_ × 2*^*n*^*, where *N* is the final cell number, *N*_0_ is the initial cell number, and *n* is the number of generations (i.e., population doubling level).

To calculate n the equation needs to be transformed:

logN=logN+0n×log2

logN-logN=0n×log2

n=(logN-logN)0÷log2

n=(logN-logN)0÷0.301

n=3.322(logN-logN)0

This equation is valid for the first passage. In every following passage, the population doubling level (i.e., number of undergone doublings) of the preceding passage needs to be added.

Thus, the cPD was calculated by the following equation:

cPD = 3.322 × (log (cell number_*harvest*_) - log (cell number_*seeded*_)) + *X*,

where *X* = population doubling level of preceding passage, i.e., cPD of inoculum.

### Projected MSC Yield

The projected MSC yield per mL collected BM was calculated as follows:

For P0:

To calculate the cell harvest per mL BM, we divided the total cell number (obtained by either DGC or device) by the volume of BM (subsequently processed by either DGC or device).

To calculate the fraction of MSCs that could be obtained from the BMCs (after either DGC or device), the number of MSCs harvested at the end of P0 was divided by the number of BMCs initially seeded.

To calculate the maximum number of MSCs that can be obtained at the end of P0, the cell harvest per mL BM was multiplied by the fraction of MSCs that could be obtained from the BMCs according to the following formula:

Cell number BMC^*a*^ ÷ volume of processed BM × cell number MSC^*b*^ ÷ cell number BMC^*c*^

a: postprocessing, b: harvest at P0, c: seeded at P0.

For the following passages, the projected MSC yield was calculated according to the formula *N* = *N*_0_ × 2*^*n*^*, where *N* is the projected MSC yield at end of the respective passage, *N*_0_ is the projected MSC yield at P0, and *n* is the cPD of the respective passage, i.e., number of cumulative doublings occurring in this passage.

### Immunophenotype

**FIGURE 1:**
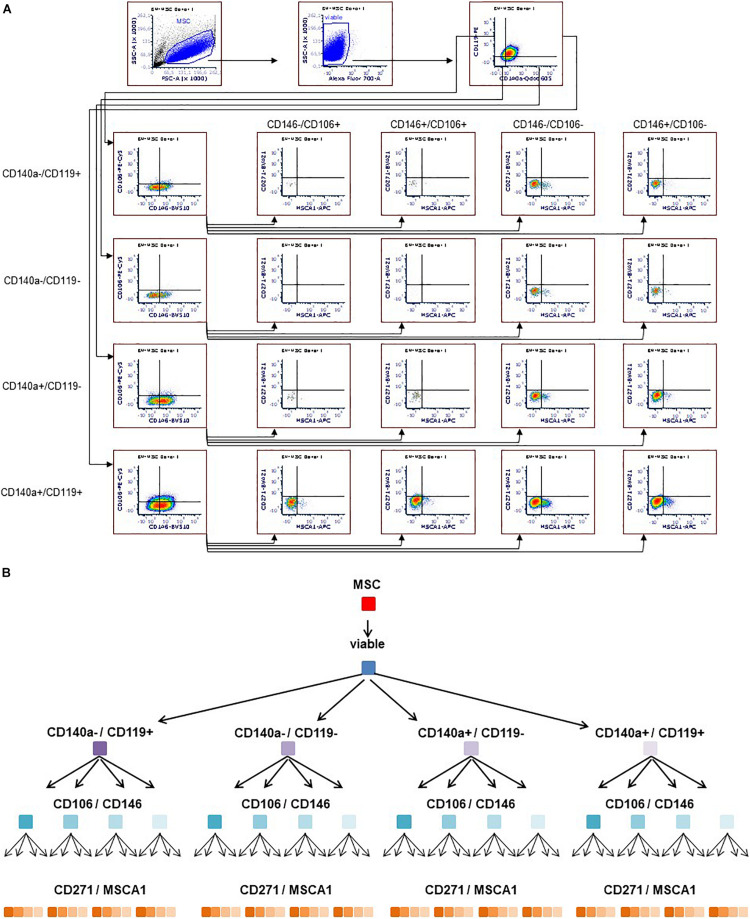
Multicolor flow cytometry gating strategy. Representative flow cytometry plots **(A)**. MSCs stained with antibodies targeting CD140a, CD119, CD146, CD106, MSCA-1, and CD271 were gated according to morphology (FSC/SSC), including viable cells only. Subsequently, the respective markers were monitored in a sequential strategy, starting with CD140a/CD119, followed by CD106/CD146, which were eventually subdivided according to CD271/MSCA-1, thus identifying 64 subpopulations **(A,B)**.

Flow cytometry was performed with LSRFortessa (Becton-Dickinson, Heidelberg, Germany). Cells were stained with 7-AAD viability dye (BioLegend, San Diego, CA, United States) and the following antibodies (all from BD Biosciences unless otherwise noted): anti–CD45-V500 (HI30), anti–CD34-PE (581), anti–CD14-FITC (M5E2, BioLegend), anti–CD19-APC (SJ25C1), anti–HLA-DR-BV421 (G46-6), anti–HLA-ABC-APC (G46-2.6), anti–CD29-BV510 (MAR4), anti–CD59-FITC (p282(H19), BioLegend), anti–CD105-PE-CF594 (266), anti–CD140b-PE (28D4), anti–CD44-AF700 (G44-26), anti–CD90-PE-Cy7 (5E10), and anti–CD73-BV605 (AD2), these comprising two separate tubes of multicolor panel (MCP) I, analyzing the MSCs with regard to the ISCT MSC criteria (Dominici et al., 2006). To identify subpopulations, MCP II was developed and established, herein staining cells with Fixable Viability Stain 700 (BD Biosciences) and the following antibodies (all from BD Biosciences unless otherwise noted): anti–CD140a-BV605 (αR1), anti–CD271-BV421 (C40-1457), anti–CD146-BV510 (P1H12), anti–CD119-PE (GIR-208), anti–CD106-PE-Cy5 (51-10C9), and anti–MSCA-1-APC (W8B2, Miltenyi Biotec). The MSCs were analyzed at P3. For multicolor flow cytometry gating strategy (see Figure 1).

### Trilineage Differentiation Potential

BM-MSCs were seeded at a density of 1,000 cells/cm^2^ at P3 and kept under standard culture conditions until reaching subconfluency. Subsequently, either adipogenic differentiation was induced using the hMSC Adipogenic BulletKit (Lonza), or osteogenic differentiation was induced using osteogenic medium composed of α-MEM/10% FBS supplemented with 1 μM dexamethasone, 50 μM ascorbic acid, and 10 mM β-glycerolphosphate (Sigma–Aldrich). After 3 weeks under differentiation conditions, cells were lineage specifically stained: lipid vacuoles in adipogenic cultures were stained with oil red O and calcium deposits of osteogenic cultures with alizarin red S, respectively. Chondrogenic differentiation of 2.5 × 10^5^ BM-MSC pellets at P3 was induced using the hMSC Chondrogenic Differentiation Medium BulletKit (Lonza), supplemented with 10 ng/mL transforming growth factor β3 (Miltenyi Biotec) as growth factor. After 4 weeks of differentiation, frozen sections of pellets were stained with Alcian blue/nuclear fast red.

### Immunomodulation and IDO-1 Production

BM-MSCs were seeded at P3 in 24-well plates at a density of 8 × 10^4^ cells per well in cell culture medium and allowed to adhere overnight.

Peripheral blood mononuclear cells (PBMNCs) were derived from buffy coats from healthy donors and labeled with 10 μM CellTrace^TM^ Violet (Thermo Fisher Scientific) according to manufacturer’s instruction. Briefly, 4 × 10^5^ labeled PBMNCs were stimulated with 10 μg/mL phytohemagglutinin (Sigma–Aldrich) in the absence or presence of MSCs in the 24-well plates in X-VIVO 10 (Lonza), corresponding to an MSC:PBMNC ratio of 1:5. After 5 days at 37°C in humidified atmosphere with 5% CO_2_, the PBMNCs were harvested and assayed for loss of fluorescent intensity by flow cytometry (LSRFortessa; Becton-Dickinson). Proliferation kinetics were analyzed using FCS Express 6 Flow Software (*De Novo* Software, Pasadena, CA, United States). The immunomodulatory potential of each MSC preparation was analyzed with PBMNCs from two different donors, in each case in duplicate.

The production of IDO-1 by BM-MSCs was stimulated in biological triplicates by tumor necrosis factor α, interferon γ1b (Miltenyi Biotec, Bergisch Gladbach, Germany), and interleukin 1β (PeproTech, Hamburg, Germany) each 20 ng/mL for 48 h. Subsequently, the cells were harvested and lysed, and IDO-1 content was analyzed in duplicate with enzyme-linked immunosorbent assay (ELISA) (Cloud-Clone Corp., Katy, TX, United States).

### Trophic Factor Production

The production of growth factors was determined in BM-MSC lysates at P3. Briefly, the BM-MSCs were lysed with ProcartaPlex^TM^ Cell Lysis Buffer and analyzed in duplicate using a ProcartaPlex Custom Panel (Thermo Fisher Scientific) according to the manufacturer’s instructions. Specifically, the kit contained beads for detection of angiopoietin-1, brain-derived neurotrophic factor (BDNF), epidermal growth factor (EGF), hepatocyte growth factor (HGF), and vascular endothelial growth factor A (VEGF-A). Analysis was performed using a LABScan 3D instrument and ProcartaPlex Analyst 1.0 Software (One Lambda/Thermo Fisher Scientific). The amount of basic fibroblast growth factor was quantified in duplicate with ELISA (RayBiotech, Norcross, GA, United States).

### *In vitro* Wound Healing

BM-MSC migration and proliferation were assessed using the *in vitro* wound healing assay at P3. Cells were seeded in 96-well plates at 1 × 10^5^ cells per well and allowed to attach overnight to form confluent monolayers. The wound was introduced across the well by removing the cells using a 96-pin WoundMaker (Essen BioScience, Welwyn Garden City, United Kingdom). Cell migration and proliferation were monitored by measuring the average wound width narrowing every 3 h using IncuCyte ZOOM (Essen BioScience).

### *In vitro* Angiogenesis

A coculture angiogenesis model (Angiogenesis Prime Kit, Essen BioScience) was used to analyze BM-MSC (P3) conditioned medium’s properties to stimulate tubule development of enhanced green fluorescent protein (eGFP^+^) human endothelial cells. Specifically, this angiogenesis model utilizes human umbilical vein endothelial cells (HUVECs) expressing eGFP in coculture with human dermal fibroblasts (NHDFs). The NHDFs support the tube formation process of the eGFP^+^ HUVECs. Tube formation can be stimulated by growth factors such as VEGF (also serving as positive control) or inhibited by adding antiangiogenic compounds (e.g., suramin, here serving as negative control). We analyzed the potential of BM-MSC conditioned medium to stimulate the tube formation of eGFP^+^ HUVECs. Briefly, BM-MSCs were cultured in 24-well plates in the IncuCyte system to near confluence, kinetically monitored by the IncuCyte ZOOM software using end point staining with propidium iodide. The supernatant of the near-confluent BM-MSC cultures was collected, quick-frozen in N_2_, and stored at -80°C until assay start. To enable comparability of the BM-MSC preparations with variable growth kinetics, the supernatant of the BM-MSC culture with the lowest cell count at harvest was defined as factor 1. The conditioned medium obtained from this culture was diluted 1:2 with assay medium of the Angiogenesis Prime Kit, and dilutions for the other samples were calculated according to the respective cell counts. The tube formation assay was performed according to manufacturer’s instructions applying conditioned medium on day 4. Tube formation was monitored in the IncuCyte system combining time lapse image acquisition with an integrated algorithm analyzing tube length. These data were exported for further statistical analysis.

### Statistical Analyses

**FIGURE 2:**
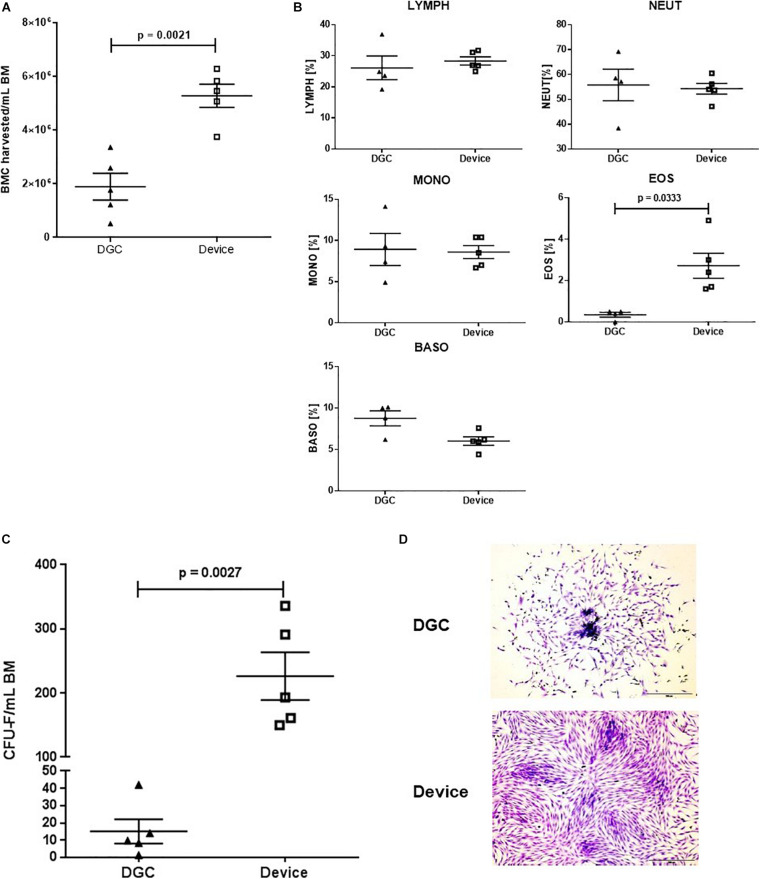
CFU-F content and growth. BMC harvested per mL BM processed with the device and by DGC **(A)** and white blood cell subsets of the cell suspensions **(B)**. Each data point represents one donor (*n* = 5 donors). Error bars: SEM. Paired *t*-test. CFU-F content per mL BM in MSCs isolated with the device and isolated by DGC at P0 **(C)**. Each data point represents one donor (n = 5 donors) as mean of two technical replicates. Error bars: SEM. Paired *t*-test. Representative microphotographs show CFU-F from both groups **(D)**. Crystal violet staining. Original magnification × 4; scale bar: 100 μm.

**FIGURE 3:**
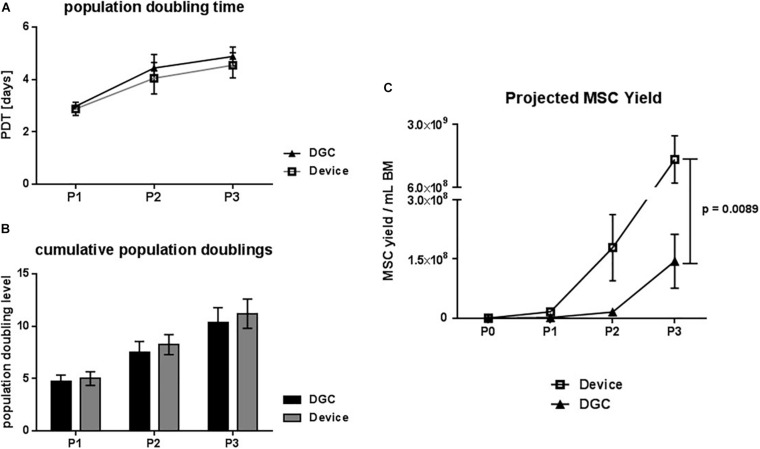
Growth kinetics. Population doubling time (PDT) in days **(A)** and cumulative population doublings **(B)** at passage (P) 1, 2, and 3. Projected MSC yield at P1–3 per mL collected BM **(C)**. Data are presented as means (*n* = 5 donors). Error bars: SEM. Two-way ANOVA. *P*-values are only shown for statistically significant comparisons.

**FIGURE 4:**
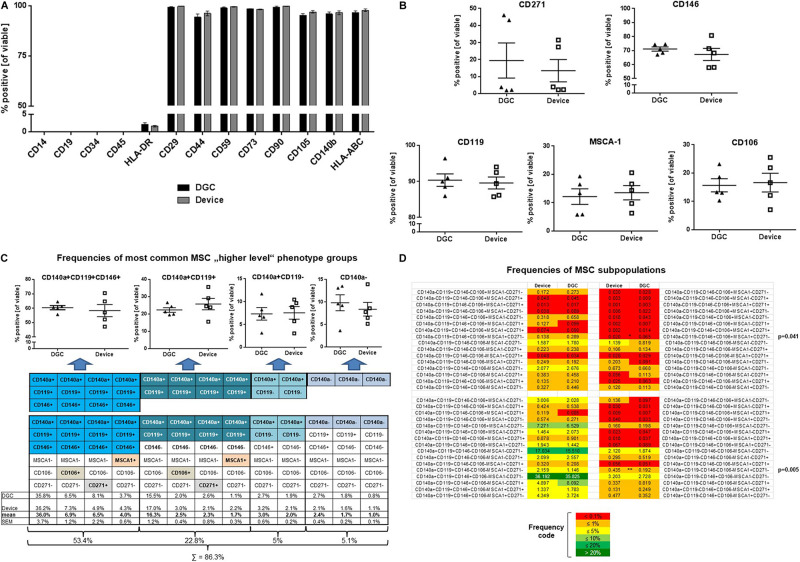
MSC surface marker phenotyping. Flow cytometry analyses of typical MSC markers, HLA class II, and hematopoietic markers at P3 **(A)**. Data are presented as means (*n* = 5 donors). Error bars: SEM. Prevalence of MSC subpopulation markers **(B)**, frequencies (in %) of “higher-level” MSC phenotype groups **(C)**, and MSC subpopulations **(D)** identified and quantified by multicolor flow cytometry at P3. Data are presented as means (*n* = 5 donors); data points in B and C represent individual donors. Error bars: SEM. One-way ANOVA. *P*-values are only shown for statistically significant comparisons.

**FIGURE 5:**
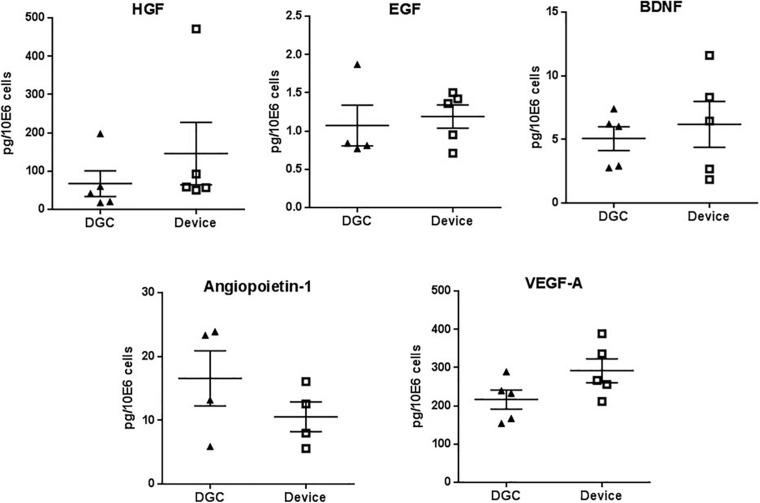
Trophic factors production. Quantification of HGF, EGF, BDNF, angiopoietin-1, and VEGF-A protein in P3 MSC lysates. Protein concentrations are calculated in picograms per million cells. Each data point represents one donor (*n* = 4–5 donors) as mean of two technical replicates. Error bars: SEM. Paired *t*-test.

Quantitative data are presented as means ± standard error of the mean (SEM) and compared with the two-tailed *t*-test (Figures 2, 5), or with analysis of variance (ANOVA) (Figures 3, 4, 7–9), using GraphPad Prism (La Jolla, CA, United States). Exact *P*-values are reported in the figures, and *P* < 0.05 was considered as statistically significant.

## Results

### MSCs Prepared With the Device Contain More Progenitor Cells, Trend to Rapid *in vitro* Proliferation, and Allow Higher Manufacturing Yields

Processing the BM with the device resulted in 2.7-fold higher numbers of BMCs that could be harvested compared to processing by DGC (Figure 2A). The white blood cell compositions of these cell suspensions were similar except for an increase of eosinophils in the device group (Figure 2B).

As expected, the CFU-F yield, indicating the amount of progenitor cells being present in the freshly processed BM (P0), was 15-fold higher in the MSCs isolated with the device compared to those isolated by DGC (Figure 2C).

Additionally, we observed that the MSCs from the device group reached earlier subconfluency in the CFU-F cultures (Figure 2D), suggesting that these cells might feature a greater proliferation potential.

Indeed, MSCs from the device group trended to faster *in vitro* growth, i.e., shorter PDTs (Figure 3A), and to more cumulative population doublings compared to the DGC group (Figure 3B). This resulted, calculated per mL of collected BM, in a more than 10-fold higher MSC yield in the device group (1.65 × 10^9^ cells) compared to the DGC group (1.44 × 10^8^ cells) at P3 (Figure 3C). Given a dose of 100 × 10^6^ cells per patient, substantially more doses could be manufactured with the device group compared to the DGC group (Table 1).

**TABLE 1:** Projection of MSC doses for 500 mL BM given a dose of 100 × 10^6^ cells per patient.

Passage	Device	DGC
1	83	9
2	895	80
3	8,250	720

### MSCs Isolated With the Device or With DGC Comprised MSC Subpopulations With Similar Phenotypes

MSCs from both groups highly expressed “typical” MSC markers such as CD29, CD44, CD59, CD73, CD90, CD105, CD140b, and HLA-ABC, whereas HLA class II expression was less than 5%, and the hematopoietic markers CD14, CD19, CD34, and CD45 could not be detected from P1 to P3 (Figure 4A and Supplementary Figure 1A).

Applying a novel designed multicolor flow cytometry comprising MSC subpopulation markers, we identified phenotype groups that included in total 64 MSC subpopulations. Over all analyzed passages (P1–P3), the prevalence of MSC subpopulation markers such as CD271, CD146, CD119, MSCA-1, and CD106 did not differ between the DGC and the device group (Figure 4B and Supplementary Figures 1B,C).

The most common phenotype groups (76.2%) in both DGC and device MSC preparations were CD140a^+^CD119^+^CD146^+^ (53.4%) and CD140a^+^CD119^+^ (22.8%) (Figure 4C). Further analyses at higher resolution identified CD140a^+^CD119^+^CD146^+^MSCA-1^–^CD106^–^CD271^–^ and CD140a^+^CD119^+^CD146^–^MSCA-1^–^CD106^–^CD271^–^ as the most frequent MSC subpopulations for both DGC-MSCs and device MSCs. Interestingly, two relatively rare subpopulations were differentially distributed between the groups, i.e., CD140a^+^CD119^–^CD146^–^MSCA-1^+^CD106^–^CD271^–^ MSCs being more present in the device group, and more CD140a^–^CD119^–^CD146^+^MSCA-1^+^CD106^+^CD271^–^ MSCs in the DGC group (Figure 4D).

### MSC Isolation Method Does Not Affect Trophic Factor Production and Differentiation Potential but May Influence Their Regenerative Functions

**FIGURE 6:**
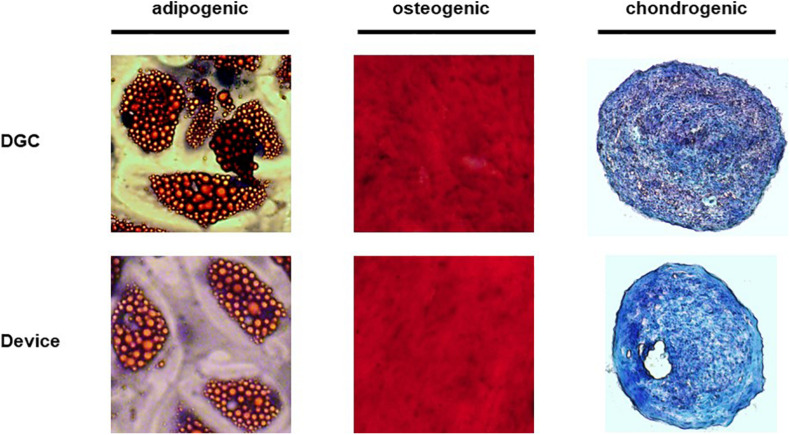
Trilineage differentiation potential. The *in vitro* differentiation potential of MSCs was assessed at P3 with Red-Oil-O staining (adipogenesis), Alizarin red staining (osteogenesis) and Alcian blue/nuclear fast red staining (chondrogenesis). Original magnification × 100 for osteogenesis and chondrogenesis, × 400 for adipogenesis.

MSCs from both groups did not differ in the production of factors that are relevant for tissue regeneration such as HGF, EGF, BDNF, angiopoietin-1, and VEGF-A (Figure 5), and we did not observe differences in the *in vitro* differentiation potential of MSCs isolated by the device or DGC (Figure 6).

**FIGURE 7:**
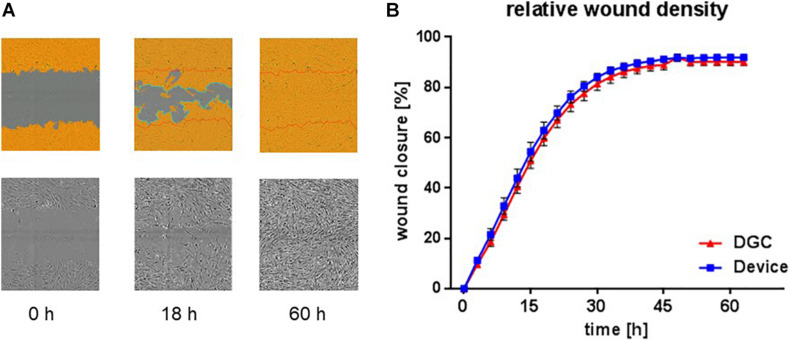
*In vitro* wound healing potential. Scratch assay to assess *in vitro* migration and proliferation, i.e., “wound healing” potential at P3. Exemplary microphotographs from life cell imaging show scratch “wound” in P3 MSC monolayer at start (0 h), proceeding (18 h), and complete closure (60 h) in% **(A)**. Images captured with 10 × objective in the IncuCyte ZOOM system. Quantification of relative wound density over time **(B)**. Each data point represents means of biological replicates (*n* = 4–5 donors). Error bars: SEM. Two-way ANOVA.

**FIGURE 8:**
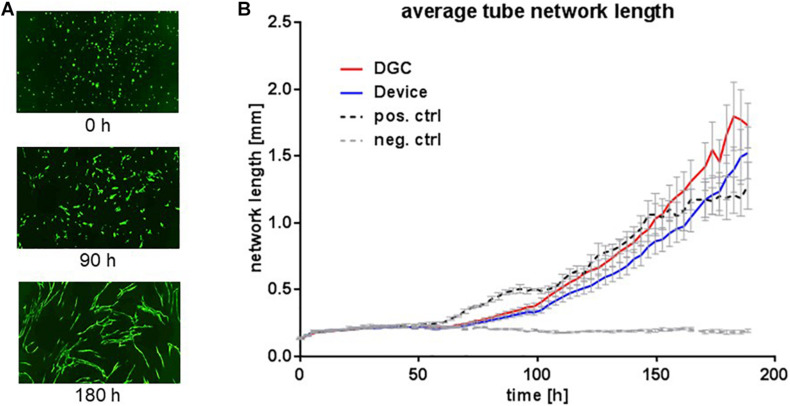
*In vitro* vascular tube formation potential. Vascular tube formation assay to assess the potential of MSCs at P3 to support *in vitro* vasculogenesis. Exemplary microphotographs from life cell imaging show eGFP^+^ HUVECs forming vascular tube networks *in vitro* at start (0 h), proceeding (90 h), and complete network formation (180 h) **(A)**. Images captured with 10 × objective in the IncuCyte ZOOM system. Quantification of average vascular tube network length in millimeters **(B)**. Each data point represents means of biological replicates (*n* = 5 donors). Error bars: SEM. Two-way ANOVA.

Tested in an *in vitro* “wound healing” assay addressing both migration and proliferation capacities and in an *in vitro* vasculogenesis assay, the MSCs showed donor-related variabilities without statistically significant differences between the groups. Yet, MSCs from the device group trended to earlier wound closure compared to the DGC group (Figure 7). In contrast, MSCs isolated by DGC showed a trend for stronger support of *in vitro* vasculogenesis as shown by vascular tube network length analysis (Figure 8).

**FIGURE 9:**
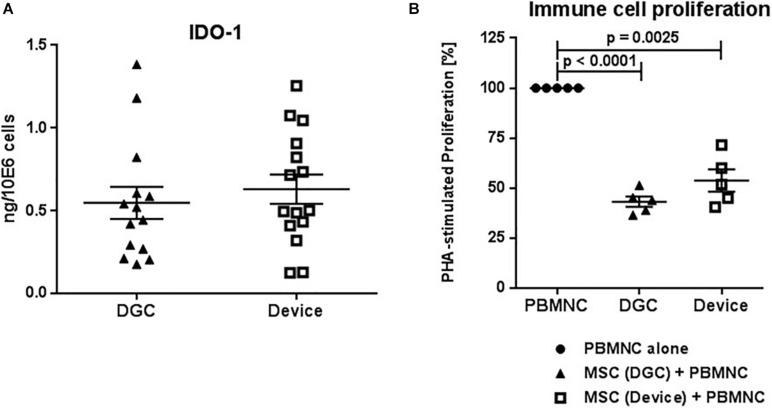
IDO-1 production and *in vitro* immunomodulation capacity. Quantification of IDO-1 protein in MSC lysates at P3 **(A)**. Protein concentrations are calculated in nanograms per million cells. Each data point represents one biological replicate (*n* = 5 donors). Error bars: SEM. Suppression of phytohemagglutinin (PHA) stimulated peripheral blood mononuclear cell (PBMNC) proliferation by P3 MSCs **(B)**. PBMNC alone vs. PBMNC + DGC MSC vs. PBMNC + device MSC. Each data point represents one biological replicate (*n* = 5 donors). Error bars: SEM. One-way ANOVA.

### MSC Isolation Method Does Neither Affect IDO-1 Production nor Their Immunomodulation Functions

MSCs from both the device group and the DGC group produced similar amounts of IDO-1 protein (Figure 9A) and, consequently, did not differ in their potential to suppress immune cell proliferation *in vitro* (Figure 9B).

## Discussion

To date, the major sources for MSC therapies manufactured are BM, adipose tissue, and umbilical cord (Schäfer et al., 2016; Kabat et al., 2020). The current dosing scale was investigated by a recent study that, analyzing more than 900 clinical trials applying MSCs from different sources and for a variety of diseases, reported median MSC doses ranging from < 50 × 10^6^ to 100 × 10^6^ cells per patient, with some trials applying doses of more than 1,000 × 10^6^ cells (Kabat et al., 2020). Even with moderate single doses (1–2 × 10^6^ cells/kg body weight) (Bader et al., 2018), the cumulative dose per patient can increase substantially because of repetitive (e.g., 3–4) applications. Interestingly, the highest median doses have been given intravenously compared to other administration routes (Kabat et al., 2020). Yet, it remains unclear if the known accumulation of MSCs in the lung after systemic application (Kabat et al., 2020; Schäfer et al., 2020). is the rationale for such high-dose regimens. This illustrates that the collection of enough high-quality starting material is critical for the production of sufficient clinical MSC doses. We reported previously that the progenitor (CFU-F) content in the initial MSC culture correlates to their proliferation capacities (Siegel et al., 2013) and thus projects the MSC yield. This was also shown in another study, where greater CFU-F recovery was achieved when using the same device we investigated here that ultimately allowed to harvest more than double the number of MSCs after two passages (Otsuru et al., 2013). Moreover, the CFU-F number corresponds to the regeneration capacity of BMCs (Hernigou et al., 2005), further supporting the relevance of the progenitor cell content for the quality and production of BM-MSC/BMC preparations. In our present study, we confirmed the superior CFU-F enrichment potential of the device compared to the DGC technology. The device harvested approximately threefold more BMCs than the DGC technology; yet, the cells of device group contained 15-fold more MSCs (precursors) demonstrating the superior potential of the device to isolate MSC. Based on the CFU-F data, we calculated a more than 10-fold higher MSC yield in the device group than in the DGC group per mL collected BM. Consequently, this could allow manufacturing substantially more MSC doses at P3, e.g., for 500 mL BM: 8,250 doses (device) vs. 720 doses (DGC), given a dose of 100 × 10^6^ cells per patient.

According to the projected cell yield (Figure 3C), substantially fewer doses could be manufactured at lower passages: 83 doses (device) vs. 9 doses (DGC) at P1, or 895 doses (device) vs. 80 doses (DGC) at P2. As outlined previously, MSCs can undergo replicative senescence leading to decrease of cell numbers and reduced immunomodulation potential, as well as to genetic instability (Bieback et al., 2012, 2019b; Loisel et al., 2017).

Thus, MSC manufacture strategies need to balance achievable cell numbers for the required doses with the minimal culture time that is needed. According to our calculations, this could be achieved at P3. Therefore, we performed the functional analyses of the MSCs at this passage to characterize the MSCs in a realistic scenario.

MSC heterogeneity exists *in vivo* (Rasini et al., 2013), and distinct MSC subpopulations can be detected in MSC preparations *in vitro* (Siegel et al., 2013; Rennert et al., 2016; Khong et al., 2019). There is emerging evidence assigning functional properties to MSC subpopulation phenotypes or to molecular signatures of MSC preparations. Surface proteins such as CD271, CD146, MSCA-1, CD106, CD119, CD140a, or distinct mRNA signatures, defining cell clusters within MSC preparations, could be assigned to MSC functions such as differentiation, tissue regeneration, immunomodulation, or wound healing (Jones and Schäfer, 2015; Wu et al., 2016; Khong et al., 2019; Kuci et al., 2019). Moreover, MSC subpopulations’ motility and adhesion capacities can affect their therapeutic efficacy (Danielyan et al., 2020; Schäfer et al., 2020).

The “adherent trapping” mechanism of the device utilizes the propensity of MSCs to favor adherence to hydrophilic and rough surfaces, and the filtering/trapping effect of the non-woven fabric could, on the one hand, decrease the shear stress of the flow (Ito et al., 2010), but may, on the other hand, exert deceleration effects on the cells. Interestingly, we detected more eosinophils in the primary cell suspensions after processing in the device group, suggesting that these cells may have a similar affinity to the filter as the MSC. Other cell types such as lymphocytes or monocytes were comparable between the groups.

To date it is unknown if, and to which extent, MSC subpopulations might be more or less enriched by the device or if the filtering process could affect the biology of these MSCs.

Thus, we aimed to investigate the possible influence of the isolation technology on the MSC subpopulation composition and their functions.

First, we analyzed the expression of “binary” MSC identity markers that have been established in the field (Dominici et al., 2006; Siegel et al., 2013). Next, we designed a novel multicolor flow cytometry panel that specifically comprised the aforementioned MSC markers identifying subpopulations that were reported as being linked to their functional properties. This sequential characterization strategy, starting from single markers, followed by “higher-level” phenotype groups to high-resolution subpopulations, allowed us to identify and quantify 64 MSC phenotypes within the MSC preparations and to compare these between the device and DGC groups. The most common “higher-level” phenotype groups in both DGC and device MSC preparations were CD140a^+^CD119^+^CD146^+^ and CD140a^+^CD119^+^ cells. Combining all six markers, we increased the resolution of our flow cytometry analysis. Hereby, we identified for the first time the CD140a^+^CD119^+^CD146^+^MSCA-1^–^CD106^–^CD271^–^ and CD140a^+^CD119^+^CD146^–^MSCA-1^–^CD106^–^CD271^–^ cells as the most frequent BM-MSC subpopulations in both device and DGC groups. Notably, the isolation technology had very little influence on the MSC phenotype composition as shown on single marker level, “higher-level” phenotype groups, or MSC subpopulations. In fact, the only statistically significant difference between device and DGC was detected for two very rare (frequency < 1%) subsets.

In the current study, we detected different frequencies neither of CD146^+^ and CD271^+^ cells nor of CD119^+^, MSCA-1^+^, or CD106^+^ cells in MSC preparations of both device and DGC groups. This does not oppose previous findings that CD146 and CD271 expression on MSCs positively correlates with the progenitor content in MSC preparations (Churchman et al., 2012; Cuthbert et al., 2012; Siegel et al., 2013), because these and our current CFU-F analyses were performed at the very early stage of the MSC culture (P1 or P0, respectively), whereas the flow cytometry analysis and the functional analyses in the current study were performed at P3, a typical culture stage of clinical MSC products (Trento et al., 2018). The MSC preparations being isolated with the device or the DGC were highly heterogeneous, but showed great similarities of the MSC subpopulation compositions. Of note, we did not detect significant differences of the CD271^+^ MSC content in both device and DGC group. This molecule (nerve growth factor receptor) was previously identified as a relevant marker of MSC populations with superior immunomodulation and *in vitro* “wound healing” potential (Kuci et al., 2010; Latifi-Pupovci et al., 2015).

Thus, we hypothesized that we would not find major differences on the functional level when MSC therapeutics are usually applied balancing the manufacture of sufficient cell numbers during a most short *ex vivo* culture time such as P2 or P3 (Trento et al., 2018). Indeed, the isolation method affected neither the trophic factors production nor the differentiation potential. Also, both groups produced comparable amounts of IDO-1, which was previously identified as a relevant mediator of the MSC immunosuppressive function (Fontaine et al., 2016). This goes in line with our observation that the immunomodulation capacities of the MSCs isolated with the device or by DGC similarly suppressed immune cell proliferation. Interestingly, we found hints that the respective isolation technique might generate MSC preparations that may promote either more migration (device) or vasculogenesis (DGC), but without statistically significant differences.

Applying the device enabled us to isolate more progenitor cells per milliliter BM compared to the mainly used BMC isolation technique (DGC), to date, which in turn has the potential to manufacture more MSC doses. We could not identify significant differences on MSC subpopulation content and their functions in the MSC preparations manufactured from BMCs isolated either by the device or by DGC. This could either be the result of qualitative similarity of BMCs of both groups and the MSCs being maintained throughout the culture up to P3, or a possible alignment process of initial differences on BMC/early passage MSC levels during *ex vivo* culture with the same media formulation and culture protocol. Here, the use of the same FBS batch as media supplement for both groups might have played a role to generate a culture environment producing highly similar MSC preparations.

Besides having the potential to isolate more MSCs per milliliter BM, the device tested here reduces the number of open steps compared to DGC. Moreover, its operation time is approximately 20 min, which is about one-fifth of that required for typical DGC protocols. This reduces contamination risks and could, by streamlining the production process and being more efficacious, also offer economic advantages in an MSC production program.

Further studies are warranted to investigate possible effects of human platelet lysate, which is increasingly replacing FBS as media supplement for MSC therapies manufacture (Bieback et al., 2019a). The presented characterization concept could have missed relevant, currently unknown, distinctive functional MSC features. To conclusively assess possible therapeutic differences, *in vivo* studies in adequate preclinical models are suggested, ideally in a multicenter study setting, as a recent study showed that the local manufacture process highly affects functional and molecular differences between MSC preparations (Stroncek et al., 2020).

## Data Availability Statement

The raw data supporting the conclusions of this article will be made available by the authors, without undue reservation.

## Ethics Statement

The study was approved by the Ethics Committee of the Goethe University Medical Center, Frankfurt, Germany (ethics committee approval #383/13).

## Author Contributions

RS designed the study, analyzed data, and wrote the manuscript. GS, AK, and A-SW performed experiments, analyzed data, and contributed to manuscript writing. ES contributed to manuscript writing. All authors contributed to the article and approved the submitted version.

## Conflict of Interest

The authors declare that the research was conducted in the absence of any commercial or financial relationships that could be construed as a potential conflict of interest.

## Supplementary Material

Supplemental Figure 1 MSC surface marker phenotyping. Flow cytometry analyses of typical MSC markers, HLA class II, and hematopoietic markers at P1 and P2 **(A)**. Data are presented as means (*n* = 2 donors). Error bars: SD. Prevalence of MSC subpopulation markers identified and quantified by multicolor flow cytometry at P1 **(B)** and P2 **(C)**. Data are presented as means (*n* = 2 donors). Error bars: SD.
