# Supplementary figures and images for: More Human BM-MSC With Similar Subpopulation Composition and Functional Characteristics Can Be Produced With a GMP-Compatible Fabric Filter System Compared to Density Gradient Technique
Source: Front Cell Dev Biol. 2021 Mar 29;9:638798. doi: 10.3389/fcell.2021.638798 (PMC8044851; doi:10.3389/fcell.2021.638798)

Supplemental Figure 1 A

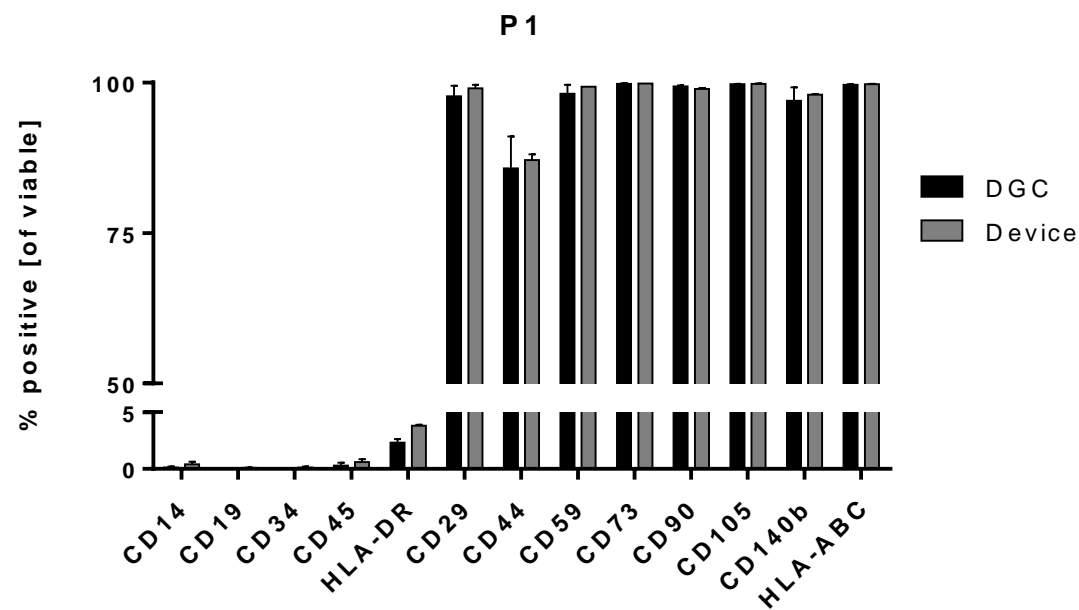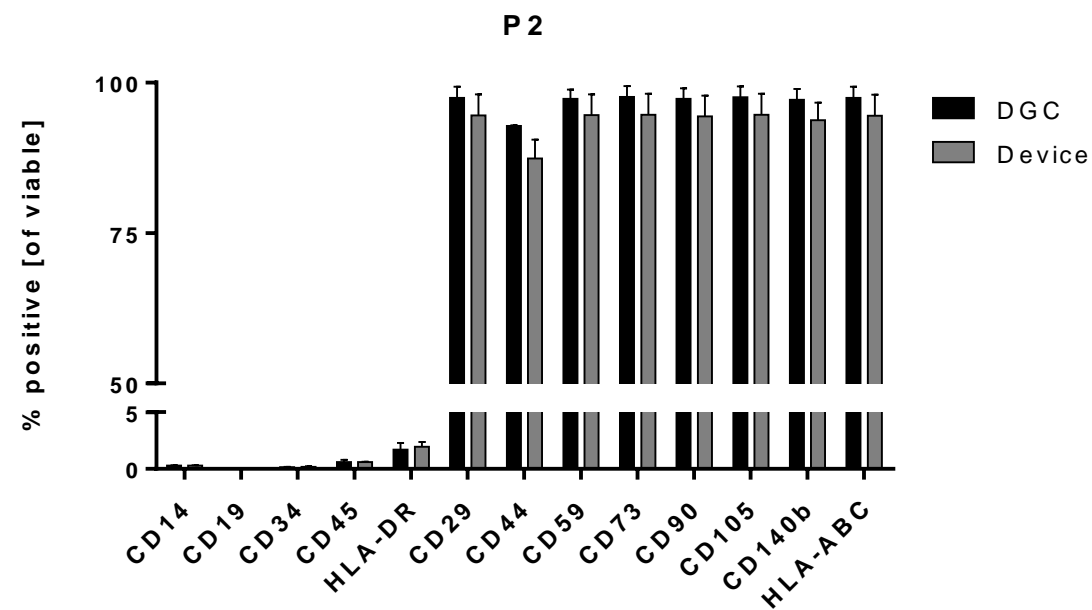

Supplemental Figure 1 B

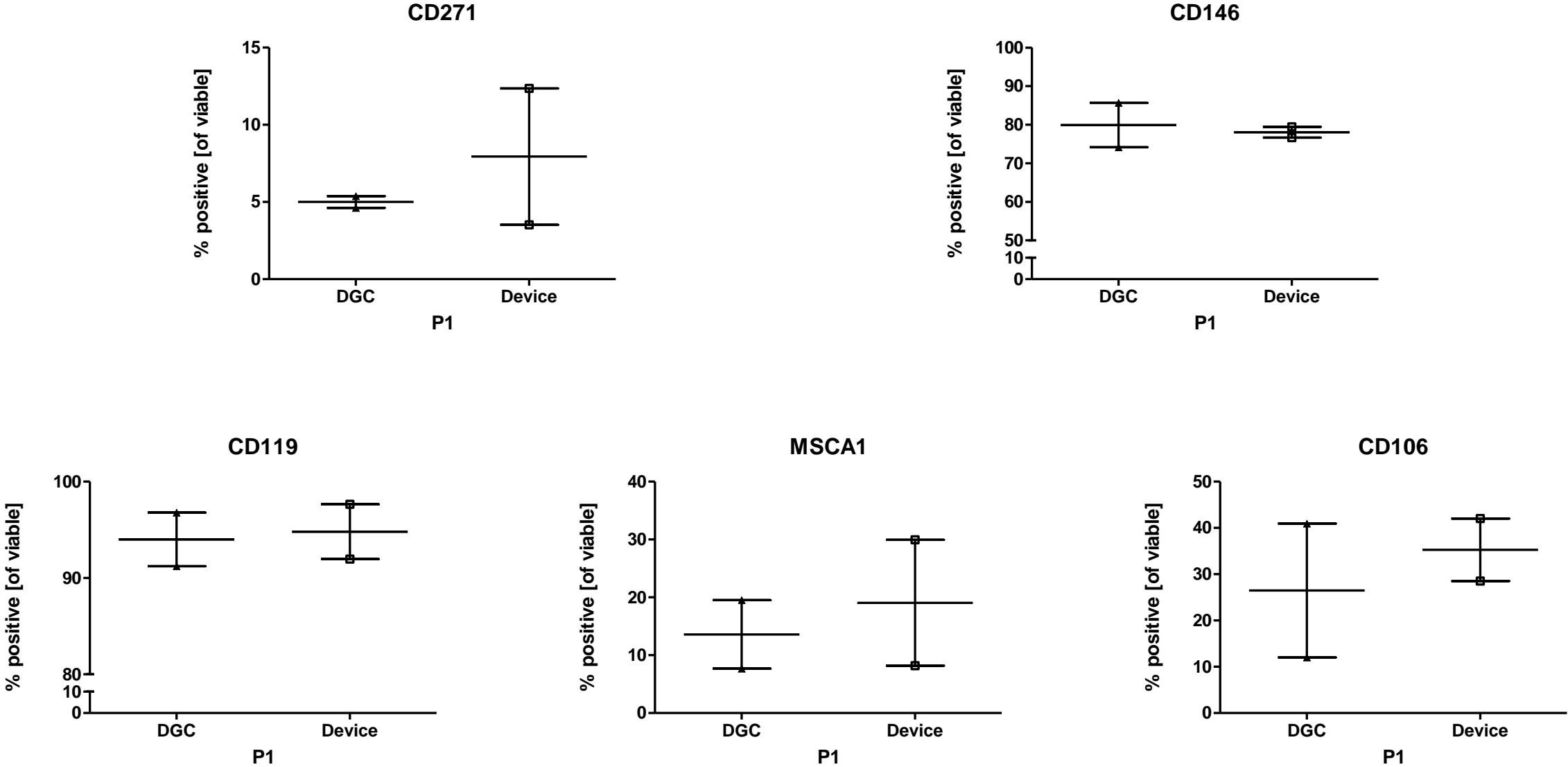

Supplemental Figure 1 C

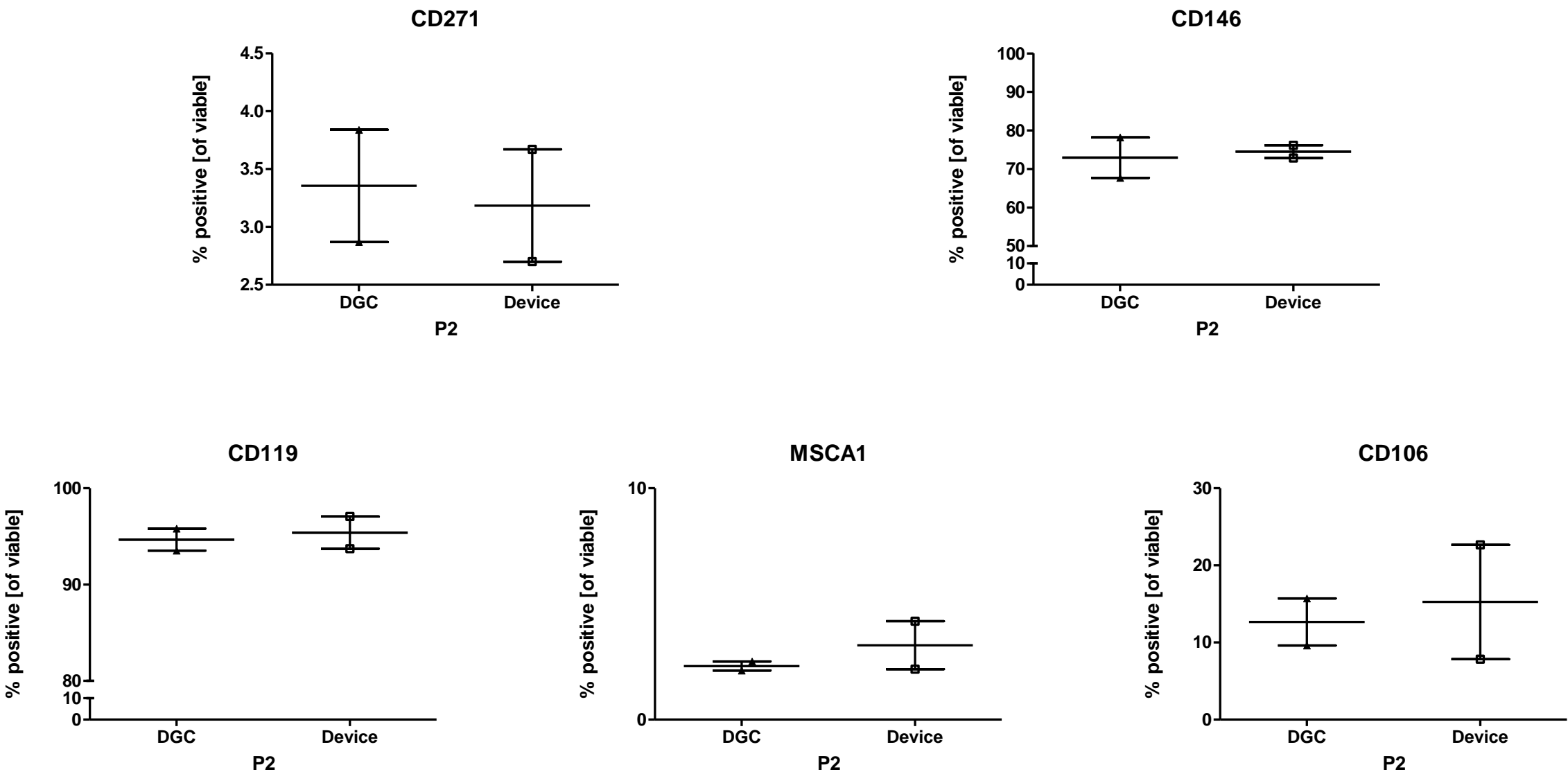

Supplement: Supplemental Figure 1 — MSC surface marker phenotyping. Flow cytometry analyses of typical MSC markers, HLA class II, and hematopoietic markers at P1 and P2 (A). Data are presented as means (n = 2 donors). Error bars: SD. Prevalence of MSC subpopulation markers identified and quantified by multicolor flow cytometry at P1 (B) and P2 (C). Data are presented as means (n = 2 donors). Error bars: SD. [file Data_Sheet_1.pdf]
